# DeepFrag-k: a fragment-based deep learning approach for protein fold recognition

**DOI:** 10.1186/s12859-020-3504-z

**Published:** 2020-11-18

**Authors:** Wessam Elhefnawy, Min Li, Jianxin Wang, Yaohang Li

**Affiliations:** 1 Department of Computer Science, Old Dominion University, Norfolk, U.S.A.; 2 Department of Computer Science, Central South University, Changsha, China

**Keywords:** Fold recognition, Protein fragments, Deep learning

## Abstract

**Background:**

One of the most essential problems in structural bioinformatics is protein fold recognition. In this paper, we design a novel deep learning architecture, so-called DeepFrag-k, which identifies fold discriminative features at fragment level to improve the accuracy of protein fold recognition. DeepFrag-k is composed of two stages: the first stage employs a multi-modal Deep Belief Network (DBN) to predict the potential structural fragments given a sequence, represented as a fragment vector, and then the second stage uses a deep convolutional neural network (CNN) to classify the fragment vector into the corresponding fold.

**Results:**

Our results show that DeepFrag-k yields 92.98*%* accuracy in predicting the top-100 most popular fragments, which can be used to generate discriminative fragment feature vectors to improve protein fold recognition.

**Conclusions:**

There is a set of fragments that can serve as structural “keywords” distinguishing between major protein folds. The deep learning architecture in DeepFrag-k is able to accurately identify these fragments as structure features to improve protein fold recognition.

## Background

The relationship between the protein amino acid sequence and its tertiary structure is revealed by protein folding. A specific protein fold describes the distinctive arrangement of secondary structure elements in the nearly-infinite conformation space, which denotes the structural characteristics of a protein molecule. A number of protein fold databases, including CATH [1] and SCOP [2], have been developed to classify these experimentally-determined protein structures according to the hierarchy of structural similarity. In the past decades, the number of identified protein sequences has dramatically increased due to high-throughput sequencing technologies; however, the number of unique structural folds remains unchanged in the past seven years [3], indicating that the protein structure universe is nearly complete. A highly accurate computational fold recognition method is a critical tool to bridge the sequence-structure gap.

Fold recognition methods can be classified into two categories: sequence alignment methods and machine learning methods [4]. The idea behind sequence alignment methods is to match a sequence or sequence profile against those with experimentally-determined structures as templates [5] to identify the most suitable fold. On the other hand, machine learning methods aim at identifying global or local features of a given sequence and then classify it into one of the known fold categories. Early machine learning fold recognition methods encompass using multi-layer perceptron and support vector machines [6]. Later, ensemble classifiers and kernel-based methods are introduced to discover correlations between sequence features to overcome the weakness of the early machine learning methods and improve the discriminability of the fold recognizers [5]. Recently, deep learning techniques have been applied to extract effective features, such as secondary structures [4] and inter-residue contacts [7], to further improve fold recognition.

In this work, we present a novel deep neural network architecture, so-called Deep-Frag-k, to classify target protein sequences into known protein folds. Unlike most of the fold recognition methods which predict folds directly from sequence and sequence-related features, Deep-Frag-k adopts a two-stage process, where a fragment vector is predicted in stage 1 and then the corresponding protein fold is predicted in stage 2. The fundamental idea in Deep-Frag-k is to predict the potential structural fragments that a target protein sequence will form [8] during folding, represented as a fragment vector, which contains highly discriminative features to distinguish a protein fold [9]. If a protein sequence is regarded as a document, the fragments can be treated as words in this document. The fragments form structural motifs, which are building blocks to assemble the protein structure. In particular, certain fragments are critical to carry out important protein functions. These fragments can be treated as “keywords” features that are able to uniquely distinguish one fold from the others.

Deep-Frag-k is composed of two stages. The first stage uses a multi-modal Deep Belief Network (DBN) to fuse multiple groups of features, including sequence composition, amino acid physicochemical properties, and evolutionary information, to precisely predict potential structure fragments for a given sequence, which are represented as a fragment vector. Then, a 1-D Convolutional Neural Network (CNN) is employed to classify the fragment vector into the appropriate fold. We evaluate DeepFrag-k on three fold recognition datasets: Ding and Dubchak (DD) [10], Extended DD (EDD) [11], and Taguchi and Gromiha (TG) [12]. Our results show that DeepFrag-k is more accurate, sensitive, and robust than the existing methods, including PFP-Pred [13], GAOEC [14], ThePFP-FunDSeqE [15], Dehzangi et al. [6, 16], MarFold [17], PFP-RFSM [18], Feng and Hu [19], Feng et al. [20], PFPA [21], Paliwal et al. [22, 23], Dehzangi et al. [24], HMMFold [25], Saini et al. [26], and Profold [27], in protein fold recognition.

## Methods

### DeepFrag-k fold recognition architecture

**Fig. 1:**
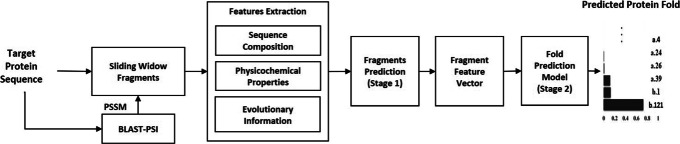
Fold recognition architecture. Two stages protein fold recognition architecture

Figure 1 presents the two-stage deep neural network architecture of DeepFrag-k. In the first stage, we predict a fragment vector representation of a target protein sequence using a fragment prediction model based on multi-modal DBN [28], which predicts the potential fragments that the target protein sequence will form during protein folding process. In particular, we focus on the top-100 most popular fragments, with 4- to 20-residue in length, described in our Frag-k fragment libraries [8, 9]. Our previous results [9] show that these fragments can be used as the structural “keywords” to effectively distinguish between major protein folds. In the multi-modal DBN, the DBNs interact with each other to learn fragment latent representation on the set of features derived from sequence composition, physicochemical properties, and evolutionary information. The output of the first stage is a fragment vector with respect to the target protein sequence. Afterwards, in the second stage, this fragment vector is fed to a 1D Convolutional Neural Network (1D-CNN) [29] classifier, as the feature vector of the target protein sequence, to predict the likeliness of the protein folds. DeepFrag-k is implemented on the Tensorflow platform. The leaky ReLU activation functions are used in the DBN and CNN layers to avoid the vanishing gradient problem and speed up training. The Adam optimization algorithm for stochastic gradient descent is adopted for training the DBN and CNN models, with learning rate of 0.0001. The training of DeepFrag-k is carried out on a GPU P40 server with 3,840 CUDA cores and 24GB GDDR5 memory.

#### Fragment prediction (Stage 1)

**Fig. 2:**
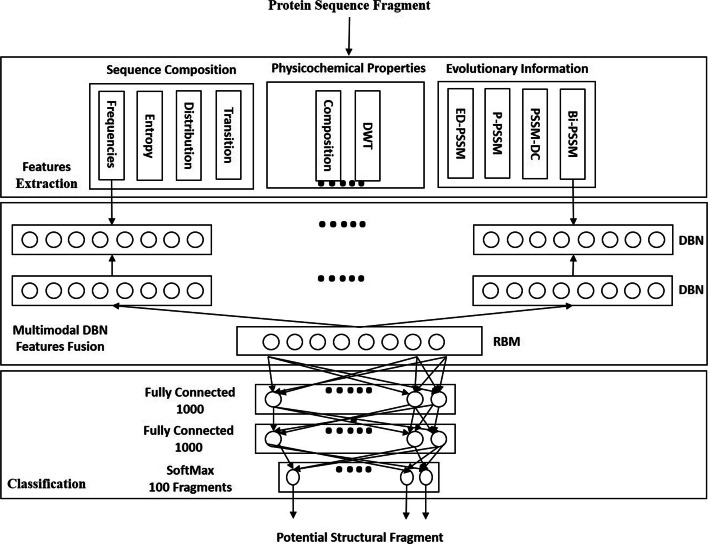
Phase I.Multi-modal DBN architecture for fragment prediction

A protein fold distinguish itself by forming certain unique secondary structures and super-secondary structure motifs, such as *β*-hairpins, short *β*-sheets, helix-loop-helix, and helix-turn-helix, which are represented as structural fragments. Correctly predicting these fragments from a given sequence can lead to effective features for fold recognition. However, the sequence features to predict fragments hold distinct statistical properties and the correlations between them are highly nonlinear [28]. For a shallow model, it is difficult to capture these correlations and form an integrated informative representation. Our fragment prediction model consists of a multimodal DBN and a fully-connected network. Our motivation for the proposed multimodal DBN is to tackle the above challenge by using an integrated representation to enhance the fragment prediction accuracy [28]. Figure 2 summarizes the framework of our proposed fragment prediction model. We use the Frag-k fragment libraries to train the fragment prediction model. First, we use the extracted sequence composition, physicochemical properties, and evolutionary information as feature groups to learn the latent representations of the top-100 Frag-k fragments. As shown in [28], the top-100 Frag-k fragments are capable of classifying major SCOP folds in high accuracy and can also be used to assemble most protein structures in high precision. The multiple feature representations learned by the DBNs are concatenated to train a Restricted Boltzmann Machine (RBM) model [28] to fuse a latent feature representation for the feature groups. Finally, two fully-connected 1,000×1,000 neural network layers followed by a SoftMax layer of 100 output nodes, representing the top-100 Frag-k fragments, are trained with these latent feature representations to generate the fragment prediction. Such layer-by-layer learning helps gradually extract the effective features from the original feature groups [30]. The multimodal DBN learns discriminative latent features as a joint distribution determined by the hidden variables of non-correlated feature groups input [28]. As a result, the hybrid framework of multi-modal learning fuses an abstraction level representation, which enables the fragment predictor to integrate different feature groups for fragments of different lengths flexibly.

The training of the fragment prediction model is performed via Stochastic Gradient Descent method. During the training process, the Frag-k fragment library, with 1,000 samples in each fragment class, is randomly split into batches, each of which contains 500 samples. In order to prevent overfitting, dropout layers are inserted after every hidden layer with 0.5 dropout rate and an early stopping strategy is employed.

#### Fold prediction (Stage 2)

**Fig. 3:**
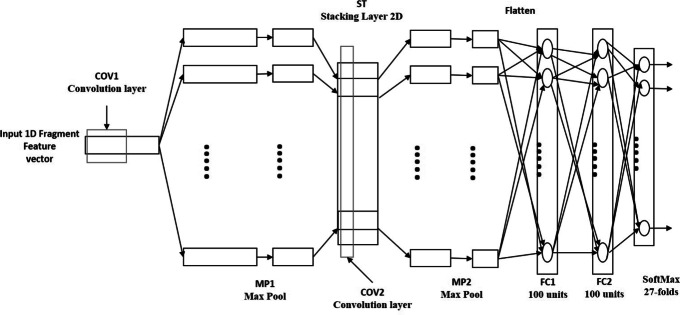
Phase II.Protein Fold Classification 1D-CNN model

The fragment feature vector generated from stage 1 is fed to a 1D-CNN architecture to predict protein fold, as shown in Fig. 3. The proposed 1D-CNN comprises two pairs of convolution and max pooling layers (COV1-MP1 and COV2-MP2), two fully-connected layers FC1 and FC2, and a SoftMax layer. Between MP1 and COV2, we include a stacking layer ST. The COV1 layer contains 10 convolution filters, producing 10 filtered versions of the fragment feature vector as output. These filtered versions are then subsampled in max pooling layer MP1. The stacking layer rearranges the output of MP1 so that a 2D stack of the generated features from COV1 is sent to the second convolution layer COV2. The convolution filters in COV2 are 2D filters, with the same height as the ST layer. The purpose of these 2D filters is to capture the relationships across the latent features produced by the convolution filters of the original fragment vector in COV1. Then the generated output is subsampled in max pooling layer MP2. In order to classify the flattened output of MP2 into corresponding folds, two fully-connected layers, FC1 and FC2, followed by a SoftMax layer are employed. We summarize the hyper-parameters for deep fold recognition architecture in Table 1.

**Table 1:** Hyperparameters for Fold Classification Architecture

Layer	Layer type	# of Units	Unit Type	Size	Stride
Input		# of fragments			
COV_1	Convolution	10	ReLU	1,10	1,1
MP_1	Max Pool			1,10	1,1
ST	Stacking				
COV_2	Convolution	100	ReLU	10,10	1,1
MP_2	Max Pool			5,5	5,5
FC_1	Fully Connected	100	ReLU		
FC_2	Fully Connected	100	ReLU		
Output	SoftMax	# of folds	Logistics		

#### Features extraction

Constructing a proper feature vector from a protein sequence is a critical step for protein fragment prediction [7]. Using multiple features extraction strategy, representing sequence, evolutionary, physicochemical information of a sequence fragment, maximizes the discriminative capability of the fold recognizer [31]. The sequence features for fragments used in DeepFrag-k include frequencies of functional groups, information entropy of amino acids and dipeptides [32], distribution of amino acids relative positions [31], and transitions of functional groups [33]. The physicochemical features include PseAAC (Pseudo Amino Acid Composition) [34] and Discrete Wavelet Transform (DWT) of hydrophobicity, flexibility, and average accessible surface area of amino acids in a fragment. The evolutionary features are described by various forms of position-specific scoring matrix (PSSM) profiles [35] including profile PSSM (P-PSSM), PSSM-Dipeptide Composition (PSSM-DC), Bi-gram PSSM (Bi-PSSM), and Evolutionary Difference-PSSM (ED-PSSM). These features are summarized in Table 2.

**Table 2:** Protein sequence features

Feature	Type	Dimension
Sequence Composition	Frequency of Function Group	10
	Information Entropy	2
	Distribution	20
	Transition	45
Physicochemical properties	Pseudo Amino Acid Composition	40
	Discrete Wavelet Transformation	42
Evolutionary Information	P-PSSM	400
	PSSM-DC	400
	Bi-Gram PSSM	400
	ED-PSSM	400

## Results

### Datasets

Three datasets, including DD [10], TG [12], and EDD [11], are used to compare the effectiveness of DeepFrag-k with existing fold recognition methods. The sequences in these datasets cover most of the sequences in the SCOP database. The DD dataset is composed of a training set and a testing set, both of which cover 27 protein folds in the SCOP database, which belong to different structural classes containing *α*, *β*, *α*/*β*, and *α*+*β*, comprehensively. The DD training set contains 311 protein sequences with ≤40*%* residue identity and the testing set contains 383 protein sequences with ≤35*%* residue identity. Additionally, the sequences in the training set have identity ≤35*%* with those in the testing dataset, ensuring to provide an unbiased performance evaluation. The TG dataset contains 1,612 protein sequences with ≤25*%* sequence identity belonging to 30 different folds in SCOP 1.73 [12]. The EDD dataset is an extended version of the DD dataset, which contains 3,418 protein sequences with ≤40*%* sequence identity [11].

### Fragment prediction model

The extracted sequence composition, physicochemical properties, and evolutionary information features of the Frag-k fragments are fed to the fragment prediction model to predict their potential corresponding fragments classes. We investigate the performance of the classifier measured by specificity, sensitivity, and accuracy, which are defined as the percentage of predicted fragment classes that are true positives, the percentage of true positives that are correctly predicted, and the fraction of fragments that are correctly classified, respectively.

**Fig. 4:**
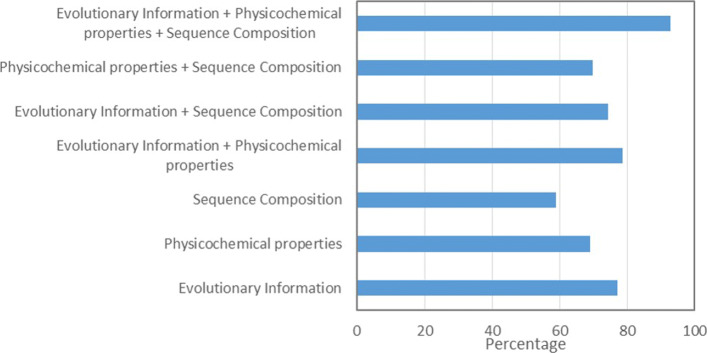
Accuracy of variable length Frag-k fragment prediction when different feature groups and their combinations are applied

We first examine the classification of sequence fragments of the same length. Figure 4 shows the accuracy, specificity, and sensitivity of the ten-fold cross-validation results for top-100 Frag-k fragment targets of length ranging from 4 to 20 residues. One can find that the prediction accuracies of longer fragments (≥10 residues) are better than those of the shorter ones, where both specificity and sensitivity are over 80*%*. This is due to the fact that the longer fragments encompass richer discriminative information. However, when the top-100 Frag-k fragments with variable lengths are used as the target classes, the prediction accuracy reaches over 90*%*, because these top-100 Frag-k fragments with variable lengths are more representative structural keywords in the protein structure universe, as we showed in our previous study [9].

**Fig. 5:**
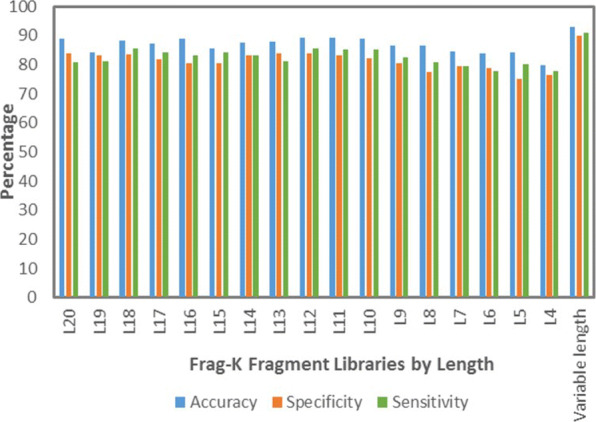
Accuracy, specificity, and sensitivity of fragment libraries models

We analyze the effectiveness of the three feature groups (Table 2) used to represent the sequence fragments on variable length Frag-k fragment prediction accuracy. We compose individual and combined sequence composition, physicochemical properties, and evolutionary information feature vectors to train the fragment prediction model showed in Fig. 2. The ten-fold cross-validation accuracy results are reported in Fig. 5. The evolutionary information plays the most important role; however, all of these feature groups contribute to the overall fragment accuracy improvements.

### Fold classification model

As shown in our previous work [9], the Frag-k fragment library with variable length achieves higher fold classification accuracy than fixed-length ones. Moreover, our results in the previous sections show that the prediction accuracy on variable length Frag-k fragments than individual fixed-length fragments. Therefore, we used the fragment vectors based on variable-length fragment predictions from the fragment prediction model for the fold recognition model.

**Fig. 6:**
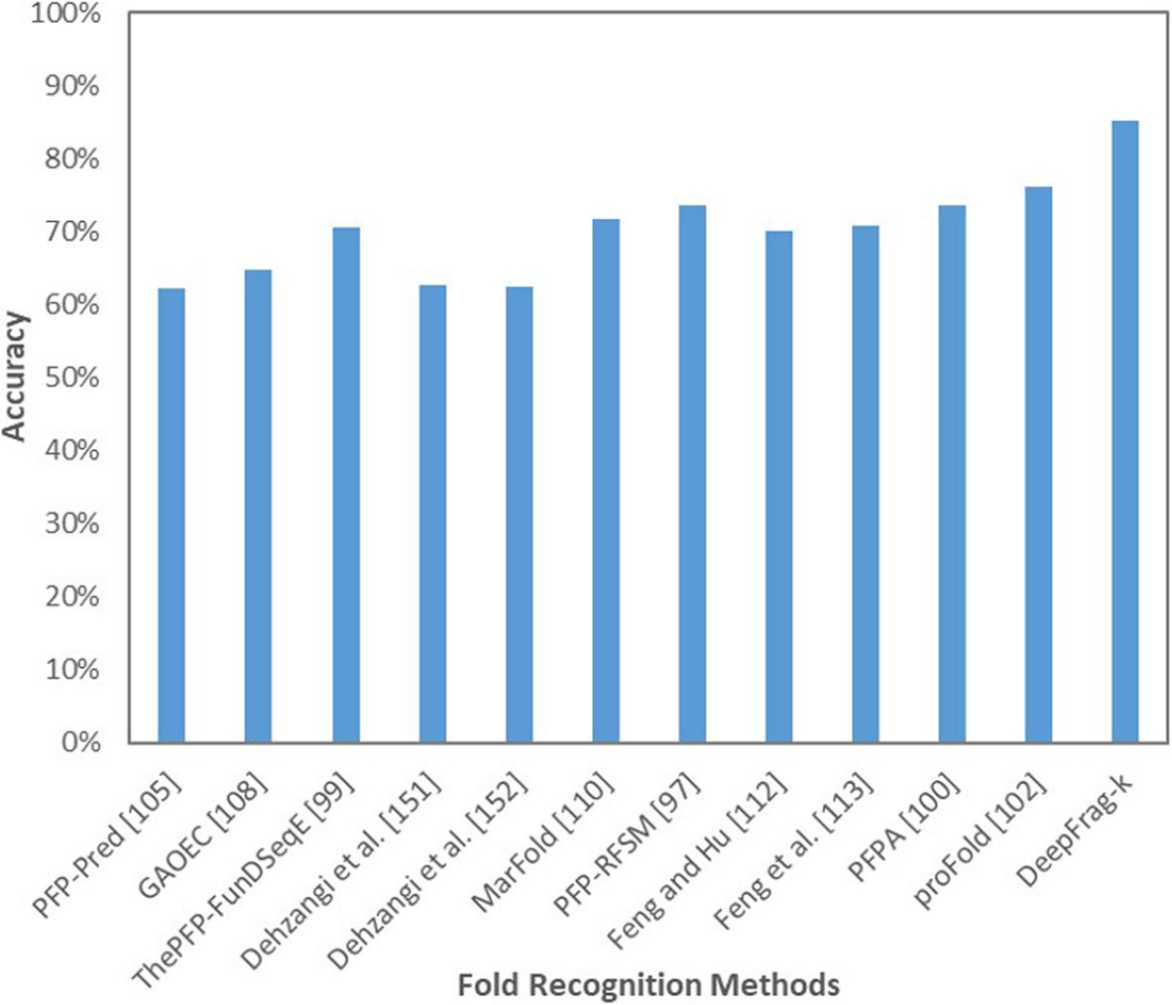
Comparison with existing fold recognition methods on DD-dataset

We use the sequences in DD, EDD, and TG datasets to evaluate the performance of DeepFrag-k. First, for a given sequence, we use a sliding window of 4 to 20 residues to consecutively segment it into a set of overlapping fragments, where gaps and non-protein residues are excluded. Figure 6 summarizes the ten-fold cross-validation results of DeepFrag-k and other fold recognition methods on the DD dataset. DeepFrag-k outperforms the other methods by yielding 85.3*%* accuracy, which is 9.1*%* higher than the second highest, proFold (76.2*%*). More detailed comparisons between DeepFrag-k and ProFold for each individual protein fold are listed in Table 3. One can find that DeepFrag-k demonstrates better fold recognition accuracy than ProFold in 18 out of 27 protein folds. It is also important to notice that DeepFrag-k shows more balanced prediction accuracy. In particular, for the folds, such as b.34, b.47, c.3, c.37, and d.15, that ProFold exhibits poor prediction results, DeepFrag-k yields significant accuracy improvements.

**Table 3:** DeepFrag-k and ProFold folds classifications accuracies for DD-dataset

#	Fold ID	Fold Name	DeepFrag-k Accuracy	ProFold Accuracy
1	a.1	Globin-like	98.0	100.0
2	a.3	Cytochrome c	95.0	100.0
3	a.4	DNA/RNA-binding 3-helical bundle	85.9	60.0
4	a.24	4-Helical up-and-down bundle	91.5	87.5
5	a.26	4-Helical cytokines	98.9	88.9
6	a.39	EF hand-like	90.8	77.8
7	b.1	Immunoglobulin-like *β*-sandwich	91.1	84.1
8	b.6	Cupredoxin-like	78.7	66.7
9	b.121	Nucleoplasmin-like/VP	91.3	92.3
10	b.29	ConA-like lectins/glucanases	76.7	66.7
11	b.34	SH3-like barrel	78.0	50.0
12	b.40	OB-Fold	80.4	68.4
13	b.42	*β*-Trefoil	89.0	100.0
14	b.47	Trypsin-like serine proteases	75.0	50.0
15	b.60	Lipocalins	90.5	100.0
16	c.1	TIM *β*/*α*-barrel	93.8	93.8
17	c.2	FAD/NAD(P)-binding domain	89.7	91.7
18	c.3	Flavodoxin-like	60.2	46.2
19	c.23	NAD(P)-binding Rossmann	90.2	85.2
20	c.37	P-loop containing NTH	79.5	50.0
21	c.47	Thioredoxin-fold	97.5	87.5
22	c.55	Ribonuclease H-like motif	75.3	58.3
23	c.69	*α*/*β*-Hydrolases	78.4	71.4
24	c.93	Periplasmic binding protein-like	92.0	100.0
25	d.15	*β*-Grasp (ubiquitin-like)	69.4	25.0
26	d.58	Ferredoxin-like	76.8	59.3
27	g.3	Knottins (small inhibitors, toxins, lectins)	88.2	96.3
Accuracy			85.3	76.2

**Fig. 7:**
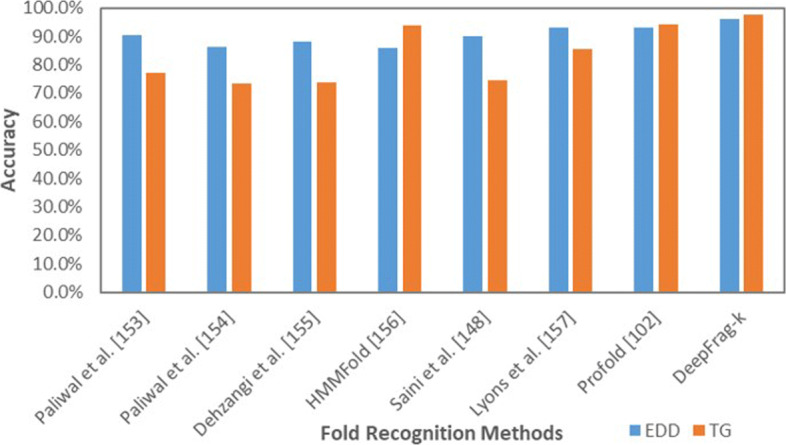
Comparing DeepFrag-k with other fold recognition methods on the TG and EDD datasets

We further evaluate the performance of DeepFrag-k on the EDD and TG datasets. The ten-fold cross-validation results in comparison with other methods are illustrated in Fig. 7. DeepFrag-k yields 96.1*%* and 97.5*%* accuracies on the EDD and TG datasets, respectively, which are higher than the other fold recognition methods. Due to significantly more samples are available in EDD and TG datasets, which is particularly helpful for our deep learning model to capture the discriminative features of the protein folds in sequence space, the DeepFrag-k yields better fold recognition accuracies in the EDD and TG datasets than that in the DD dataset.

**Fig. 8:**
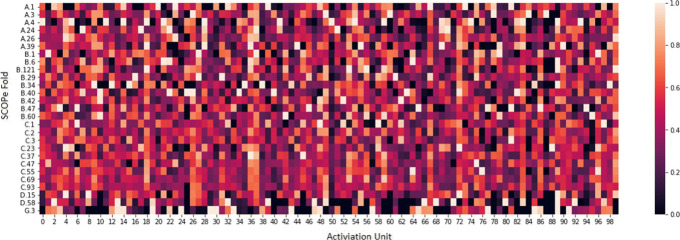
Class activation map for EDD fold classification in DeepFrag-k

Figure 8 depicts the Class Activation Map (CAM) [36] of DeepFrag-k on the EDD dataset to show how protein folds classified based on the fragment feature vectors from the protein sequences. The activation units that are most discriminative to fold classifications are identified, which are highly weighted. The combination of these class-specific units guides DeepFrag-k in distinguishing each fold. One can observe that the fold classification model makes use of more activation units to classify *α*/*β* or *α*+*β* proteins (C.1 to C.93), when compared to all *α* (A.1 to A.39) and all *β* proteins (B.1 to B.60). However, in folds of small proteins, such as G.3, only a few activation units are effective in the fold recognition process.

## Discussions

In our previous work [9], we develop a protein structural fragment library (Frag-k), composed of about 400 backbone fragments ranging from 4 to 20 residues, as the structural “keywords” in the protein structure universe. A structure dictionary using these fragments as keywords can classify the major protein folds with high accuracy. The success of DeepFrag-k is due to identifying these keywords with high precision as structural features that are effective for fold recognition. The deep learning architecture in DeepFrag-k plays an important role in accurately identifying these fragments.

The current version of DeepFrag-k has its limitations. The CNN used in the Stage 2 training of DeepFrag-k is effective in capturing local interaction patterns between fragments, but have difficulty in learning their high-order, long-range interactions, which are essential to form stable spatial structures. This problem may be addressed by incorporating deep learning techniques, such as Recurrent Neural Network (RNN), that can learn sequence data as time series and capture long-range correlations.

## Conclusions and future research directions

In this paper, we design DeepFrag-k, a two-stage deep learning neural network architecture, for fold recognition. The fragment prediction stage derives effective fragment feature vectors by fusing sequence composition, physicochemical properties, and evolutionary information features groups of sequence fragments to the fold recognition stage. Due to the highly discriminative capability of the fragment feature vectors, DeepFrag-k yields significant accuracy enhancement compared to other fold recognition methods on the DD, EDD, and TG datasets.

We will investigate using RNN to capture high-order, long-range interactions between structural fragments to further improve DeepFrag-k. Moreover, the features derived in DeepFrag-k are based on sequence fragments. They can be incorporated with other sequence or structure features, such as inter-residue interactions [7], to further improve fold recognition. Moreover, accurate fold recognition allows cooperatively fitting sequences into known three-dimensional folds, increasing the success rate by detecting very remote homologies. The recognized folds can be used as high-quality templates to predict tertiary structures in high resolutions. These will be our future research directions.

## Data Availability

The DeepFrag-k package can be downloaded at https://github.com/yaohangli/DeepFragK.

## Abbreviations

CNN: Convolutional Neural Network
RNN: Recurrent Neural Network
DBN: Deep Belief Network
RBM: Restricted Boltzmann Machine
PSSM: Position-Specific Scoring Matrix
DWT: Discrete Wavelet Transform
PseACC: Pseudo Amino Acid Composition
DD: Ding and Dubchak dataset
EDD: extended Ding and Dubchak dataset
TG: Taguchi and Gromiha dataset

## References

[1] Orengo C, Michie A, Jones S, Jones D, Swindells M, Thornton J (1997). Cath–a hierarchic classification of protein domain structures. Structure.

[2] Murzin A, Brenner S, Hubbard T, Chothia C (1995). Scop: a structural classification of proteins database for the investigation of sequences and structures. J Mol Biol.

[3] Growth Of unique folds per year as defined by SCOP. http://www.rcsb.org/pdb/statistics/contentGrowthChart.do?content=fold-scop. Accessed 22 Feb 2019.

[4] Yang Y, Heffernan R, Paliwal K, Lyons J, Dehzangi A, Sharma A, Wang J, Sattar A, Zhou Y (2017). Spider2: A package to predict secondary structure, accessible surface area, and main-chain torsional angles by deep neural networks. Methods Mol Biol.

[5] Lyons J, Paliwal K, Dehzangi A, Heffernan R, Tsunoda T, Sharma A (2016). Protein fold recognition using hmm-hmm alignment and dynamic programming. J Theor Biol.

[6] Dehzangi A, Phon-Amnuaisuk S, Dehzangi O (2010). Using random forest for protein fold prediction problem: An empirical study. J Inf Sci Eng.

[7] Zhu J, Zhang H, Li S, Wang C, Kong L, Sun S, Zheng W, Bu D (2017). Improving protein fold recognition by extracting fold-specific features from predicted residue-residue contacts. Bioinformatics.

[8] Elhefnawy W, Li M, Wang J, Li Y (2017). Construction of protein backbone fragments libraries on large protein sets using a randomized spectral clustering algorithm. International Symposium on Bioinformatics Research and Applications (ISBRA).

[9] Elhefnawy W, Li M, Wang J-X, Li Y (2019). Decoding the structural keywords in protein structure universe. j. of computer science and technology. J Comput Sci Technol.

[10] Tan A, Gilbert D, Deville Y (2003). Multi-class protein fold classification using a new ensemble machine learning approach. Genome Inform.

[11] Dong Q, Zhou S, Guan J (2009). A new taxonomy-based protein fold recognition approach based on autocross-covariance transformation. Bioinformatics.

[12] Taguchi Y, Gromiha M (2007). Application of amino acid occurrence for discriminating different folding types of globular proteins. BMC Bioinformatics.

[13] Shen H, Chou K (2006). Ensemble classifier for protein fold pattern recognition. Bioinformatics.

[14] Guo X, Gao X (2008). A novel hierarchical ensemble classifier for protein fold recognition. Protein Eng Des Sel.

[15] Shen H, Chou K (2009). Predicting protein fold pattern with functional domain and sequential evolution information. J Theor Biol.

[16] Dehzangi A, Phon-Amnuaisuk S, Manafi M, Safa S (2010). Using rotation forest for protein fold prediction problem: An empirical study. European Conference on Evolutionary Computation, Machine Learning and Data Mining in Bioinformatics.

[17] Yang T, Kecman V, Cao L, Zhang C, Huang J (2011). Margin-based ensemble classifier for protein fold recognition. Expert Systems with App.

[18] Li J, Wu J, Chen K (2013). Pfp-rfsm: Protein fold prediction by using random forests and sequence motifs. J Biomed Sci Eng.

[19] Feng Z, Hu X (2014). Recognition of 27-class protein folds by adding the interaction of segments and motif information. Biomed Res Int.

[20] Feng Z, Hu X, Jiang Z, Song H, Ashraf M (2016). The recognition of multi-class protein folds by adding average chemical shifts of secondary structure elements. Saudi J Biol Sci.

[21] Wei L, Liao M, Gao X, Zou Q (2015). Enhanced protein fold prediction method through a novel feature extraction technique. IEEE Trans Nanobioscience.

[22] Paliwal K, Sharma A, Lyons J, Dehzangi A (2014). A tri-gram based feature extraction technique using linear probabilities of position specific scoring matrix for protein fold recognition. IEEE Trans Nanobioscience.

[23] Paliwal K, Sharma A, Lyons J, Dehzangi A (2014). Improving protein fold recognition using the amalgamation of evolutionary-based and structural based information. BMC Bioinformatics.

[24] Dehzangi A, Paliwal K, Lyons J, Sharma A, Sattar A (2014). A segmentation-based method to extract structural and evolutionary features for protein fold recognition. IEEE/ACM Trans Comput Biol Bioinform.

[25] Lyons J, Dehzangi A, Heffernan R, Yang Y, Zhou Y, Sharma A, Paliwal K (2015). Advancing the accuracy of protein fold recognition by utilizing profiles from hidden markov models. IEEE Trans Nanobioscience.

[26] Saini H, Raicar G, Sharma A, Lal S, Dehzangi A, Lyons J, Paliwal K, Imoto S, Miyano S (2015). Probabilistic expression of spatially varied amino acid dimers into general form of chous pseudo amino acid composition for protein fold recognition. J Theor Biol.

[27] Chen D, Tian X, Zhou B, Gao J (2016). Profold: Protein fold classification with additional structural features and a novel ensemble classifier. Biomed Res Int.

[28] Srivastava N, Salakhutdinov R (2014). Multimodal learning with deep boltzmann machines. Adv Neural Inf Process Syst.

[29] Min S, Lee B, Yoon S (2017). Deep learning in bioinformatics. Brief Bioinform.

[30] Goodfellow I, Bengio Y, Courville A. Deep learning. Adaptive computation and machine learning series. MIT press; 2016.

[31] Dayhoff M, Schwartz R, Orcutt B. A model of evolutionary change in proteins. Atlas of protein sequence and structure. 1978; 22:345.

[32] Strait B, Dewey T (1996). The shannon information entropy of protein sequences. Biophys J.

[33] Dubchak I, Muchnik I, Holbrook S, Kim S (1995). Prediction of protein folding class using global description of amino acid sequence. Proc Natl Acad Sci U S A.

[34] Shen H, Chou K (2008). Pseaac: a flexible web server for generating various kinds of protein pseudo amino acid composition. Anal Biochem.

[35] Altschul S, Madden T, Schaffer A, Zhang J, Zhang Z, Miller W, Lipman D (1997). Gapped blast and psi-blast: a new generation of protein database search programs. Nucleic Acids Res.

[36] Zhou B, Khosla A, Lapedriza A, Oliva A, Torralba A. Object detectors emerge in deep scene cnns. arXiv 1412.6856. 2014.

